# Method for immobilization of living and synthetic cells for high-resolution imaging and single-particle tracking

**DOI:** 10.1038/s41598-018-32166-y

**Published:** 2018-09-13

**Authors:** Łukasz Syga, Dian Spakman, Christiaan M. Punter, Bert Poolman

**Affiliations:** Department of Biochemistry University of Groningen Nijenborgh 4, 9747 AG Groningen, The Netherlands

## Abstract

Super-resolution imaging and single-particle tracking require cells to be immobile as any movement reduces the resolution of the measurements. Here, we present a method based on APTES-glutaraldehyde coating of glass surfaces to immobilize cells without compromising their growth. Our method of immobilization is compatible with *Saccharomyces cerevisiae*, *Escherichia coli*, and synthetic cells (here, giant-unilamellar vesicles). The method introduces minimal background fluorescence and is suitable for imaging of single particles at high resolution. With *S. cerevisiae* we benchmarked the method against the commonly used concanavalin A approach. We show by total internal reflection fluorescence microscopy that modifying surfaces with ConA introduces artifacts close to the glass surface, which are not present when immobilizing with the APTES-glutaraldehyde method. We demonstrate validity of the method by measuring the diffusion of membrane proteins in yeast with single-particle tracking and of lipids in giant-unilamellar vesicles with fluorescence recovery after photobleaching. Importantly, the physical properties and shape of the fragile GUVs are not affected upon binding to APTES-glutaraldehyde coated glass. The APTES-glutaraldehyde is a generic method of immobilization that should work with any cell or synthetic system that has primary amines on the surface.

## Introduction

Fluorescence microscopy is a common method for studies of biological processes. Information about the localization, interactions and structure of macromolecules and sub-cellular organization of the cell can be obtained. Synthetic and genetically encoded fluorescent probes have been developed to label DNA, RNA, proteins, and lipids, allowing visualization of these major components of the cell^1–3^. Fluorescence microscopy is suited for imaging of living cells due to simpler and milder sample preparation, compared to other single-cell techniques like Atomic Force Microscopy or Electron Microscopy. Additionally, the specificity of fluorescent labeling allows for easy identification of the object of the study. The main disadvantage of conventional light microscopy is its inability to resolve fluorescent signals that are separated by less than 0.61*λ/NA, where λ is the wavelength of the light and NA is the numerical aperture of the microscope. This results in a maximal resolution of around 200 nm for commonly used setups with high NA optics. However, it is possible to localize molecules with much higher accuracy by limiting the number of fluorescent molecules emitting at the same time. Single fluorophores can be localized with tens of nanometer accuracy when each diffraction limited peak contains only one source of fluorescence^4^. The localization accuracy of a fluorophore depends on the number of photons emitted^5^ and is typically much better for dyes than for fluorescent proteins. The development of super-resolution techniques^6,7^ such as photoactivated localization microscopy (PALM) and stochastic reconstruction microscopy (STORM) is based on photoswitchable fluorescent proteins and dyes, respectively. Fluorophores are excited one by one after which they enter a dark state; by recording the position of individual molecules over time, a high-resolution image of substructures in the cell can be obtained. Dynamic information of the cell can be obtained by single-particle tracking (SPT)^4^. Here, the trajectories of individual molecules are traced for a long period of time. Both high-resolution imaging and tracking of fluorescent molecules require cells to be relatively immobile, as any movement of the cell will decrease the localization accuracy of the fluorophore. With relatively immobile we mean that the object (*e.g*. a cell) does not detectibly move on the timescale of the measurements.

The perfect immobilization method should have three main features: (i) no or minimal movement of the cell, (ii) being benign to the cells, and (iii) give rise to minimal fluorescence background. Depending on the type of experiment performed, each of the three features will have a different priority. For example, total internal reflection fluorescence (TIRF) microscopy exploits the property of an induced evanescent wave in a region of a few hundred nanometers, immediately adjacent to the interface between two media having different refractive indices^8^. The excitation power decays exponentially with the distance from the surface, reducing background coming from fluorescent molecules above the excited area. Since the glass surface is excited with more power than the sample, it is extremely important that the immobilization method does not cause background fluorescence. SPT measurements of *e.g*. membrane proteins^9^ are typically performed over long periods of time and require cells to be immobile for the duration of the experiment. In single-particle tracking or super-resolution imaging experiments the fluorophores can be localized with an accuracy of 20–50 nm. Any movement of a cell will increase error of the measurements for which a correction is often not possible.

Various methods of cell immobilization have been described. Cells can be captured in microfluidic devices^10–12^, but those devices are only suitable for tracking large changes in cell morphology^13^, or for the global monitoring of protein expression^12^. The cells are trapped at a specific location in the device but still have some freedom to move. To minimize movements, high pressures have been applied with the risk of affecting the physiology of the cells. An alternative approach to immobilize cells is to bind them to the surface of a glass slide. One such method is based on the treatment of glass surfaces with (3-aminopropyl)triethoxysilane (APTES)^14^. Highly negatively charged cell surfaces, like those of *Escherichia coli*, will be immobilized due to a strong electrostatic interaction. However, a method based on electrostatic interactions does not work for all cells, including *Saccharomyces cerevisiae*. In those cases, the carbohydrate-binding protein concanavalin A (ConA)^15^, a lectin, has been used for immobilization.

**Figure 1:**
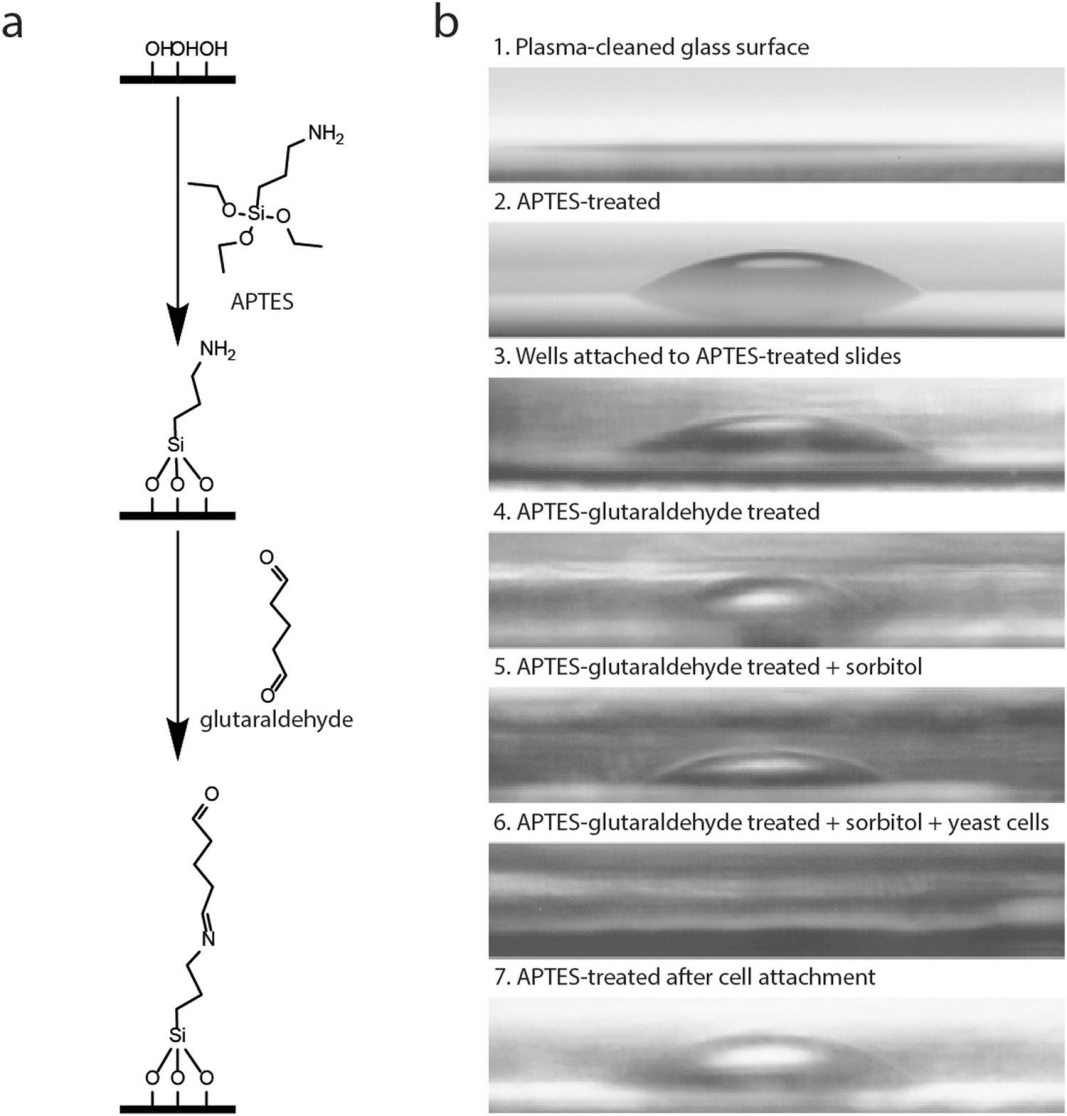
Modification of coverslips with APTES-glutaraldehyde. Panel a shows the modification of the glass. The glass surface reacts with the APTES, leaving a free primary amine on the surface. Glutaraldehyde reacts with the primary amine, leaving a second aldehyde group to react with an amine on the surface of the cell (e.g. lysine in proteins on the cell surface or lipids with phosphatidylethanolamine headgroup). Panel b shows contact angle images made at each step. Glass after plasma cleaning (image 1) and slides with cells attached (image 6) are too hydrophilic for water drop formation.

In this work, we found that cover slides prepared for immobilization with ConA show fluorescent loci when excited with a 561 nm laser. The intensity of fluorescence is not high, but it is a problem in TIRF measurements where the excitation power decays exponentially with distance and is highest near the surface. Consequently, fluorescent signals coming from the glass surface convolute the observations of fluorescent proteins inside the cell. We developed a generic method to immobilize cells for super-resolution imaging and single particle tracking measurements based on APTES coating of glass slides, followed by glutaraldehyde treatment and subsequent reaction with amines on the surface of the cells (Fig. 1a). A similar approach has been used to immobilize enzymes^16–18^ and cellular microreactors^19^. Our method offers low fluorescent background, quick attachment of different types of cells, and no visible movement of the cells over hours. The cells can be imaged in any solution and grow in the proper media during the imaging.

## Results

### Modification of the glass surface

The modification of the glass cover slides by APTES *w/wo* glutaraldehyde was investigated with contact angle measurements; details of the method are described in the Methods section under “**Preparation and characterization of coverslips”**. The plastic wells used for the immobilization of cells precluded exact determination of the contact angle, because imperfections of the plastic obscured the view. We therefore show the images of water droplets rather than actual values (Fig. 1b). First, the contact angle of plasma-cleaned coverslips was analyzed, and as expected the hydrophilic nature of the surface prevents formation of the droplet (image 1). With APTES, a clear droplet is formed (image 2), confirming the hydrophobic nature of the coating, which is not affected by attachment of the plastic wells (image 3). We then treated the wells for 30 min with glutaraldehyde, after which they were cleaned with water (image 4). A 150 mM sorbitol solution, which is equiosmolar to the growth medium used for *Saccharomyces cerevisiae*, was added to a well without (image 5) or with cells (image 6). We see that modification of the slides with glutaraldehyde increases the contact angle, but the effect is diminished in the presence of sorbitol. Furthermore, we observed that the hydrophilic groups on the surface of the *S. cerevisiae* cells prevent formation of a droplet on the glass surface, while time and handling of the coverslip had only minimal effect on the contact angle measurements (image 7).

### Immobilization of *S. cerevisiae* and optimization of the method

**Figure 2:**
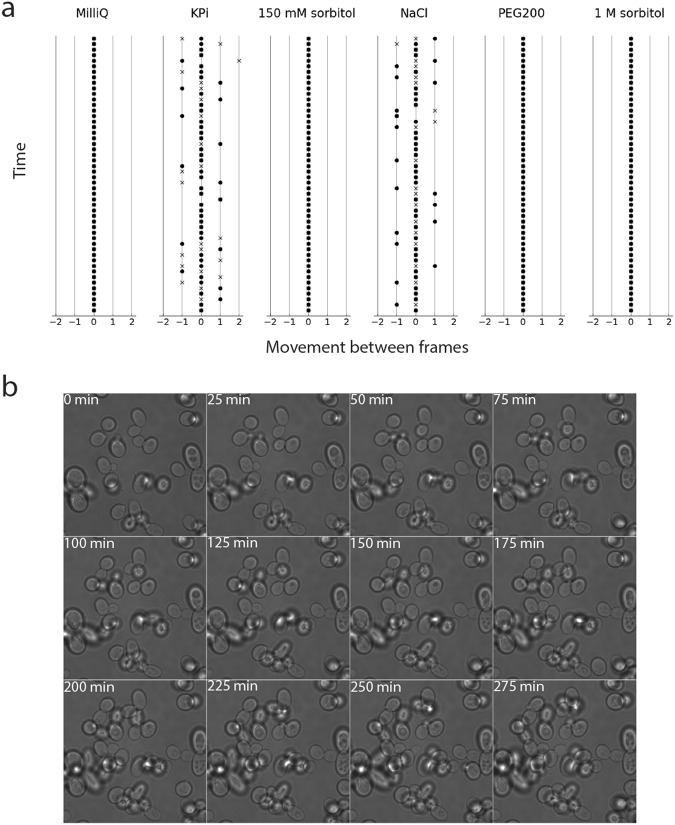
Immobilization of *S. cerevisiae* on APTES-glutaraldehyde treated coverslips. Panel a shows movement of the cells that were attached to the slides in different solutions, as indicated above the panels: The final concentrations in the attachment solutions were MilliQ, 75 mM KPi, 150 mM or 1 M sorbitol, 75 mM NaCl, and 150 mM PEG. The x-axis of the graphs shows the detected movement between two consecutive frames measured in pixels (dots represent movement along x-axis, crosses along y-axis). Multiple frames are plotted on the y-axis. Panel b shows growth of *S. cerevisiae*. The cells were immobilized in the presence of 150 mM sorbitol, after which the sorbitol was replaced by synthetic drop-out medium containing 2% [w/v] D-raffinose without uracil.

Next, we optimized the conditions for the immobilization of the yeast *Saccharomyces cerevisiae*. From previous work, we knew that yeast cells could be attached to APTES-glutaraldehyde treated coverslips using ddH_2_O as the attaching solution^9^. However, suspension of cells in ddH_2_O results in an osmotic downshift, which should be avoided if possible. Immobilization of the cells in growth media is not possible because high amounts of amino acids and others primary amines are present and these will react with the glutaraldehyde on the coverslips. We wanted the attaching solutions to be close to physiological conditions, so we started with solutions that have a pH and osmolality similar to that of the growth media. To investigate which solution works best, we used optical microscopy and monitored the movement of cells over time (Fig. 2a, Supplementary Movie 1). We tested 75 mM potassium phosphate (KPi), pH 6.5, and 75 mM NaCl but observed that the presence of salts affects the immobilization of yeast cells to APTES-glutaraldehyde treated coverslips, presumably by shielding primary amine groups (*vide infra*). Next, we tested sorbitol and PEG200 at 150 mM up to 1 M; these non-ionic solutions allow immobilization of yeast cells, but with PEG200 a subpopulation of the cells remained mobile. We continued with 150 mM sorbitol as it gave us the best results. Sorbitol is commonly used in studies of yeast, either to protect spheroplasts from lysing, or at high concentrations as a shocking agent^20–23^.

**Figure 3:**
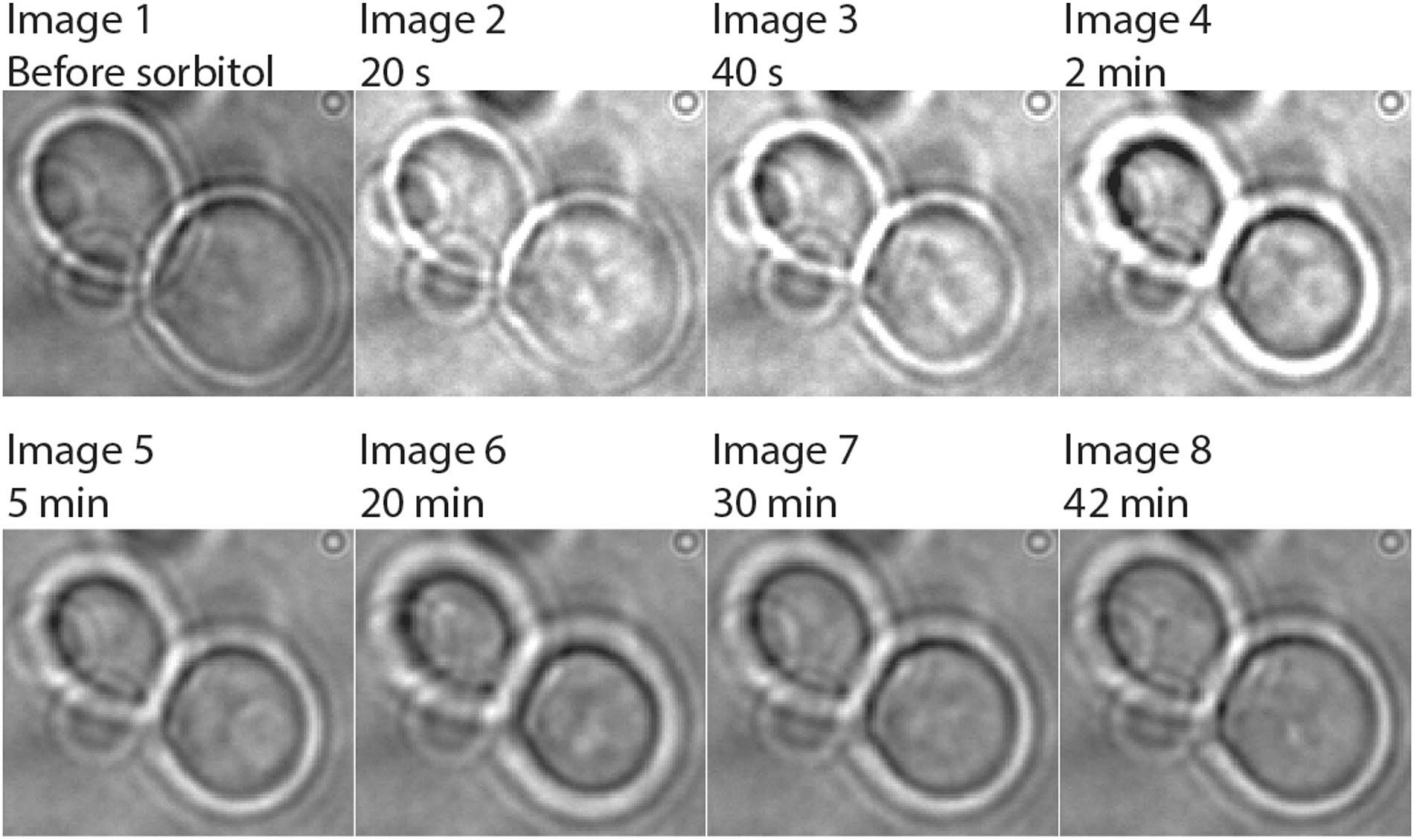
Effect of hyper-osmotic stress on volume of *S. cerevisiae*. Cells were immobilized on APTES-glutaraldehyde modified coverslips. They were imaged in media allowing growth prior to the osmotic shock. Then, media with sorbitol was added to a final concentration of sorbitol of 1 M. Upon addition of sorbitol the cells rapidly shrink (images 2–4), after which they adapt and recover their volume over a period of approximately 30 min.

Importantly, cells immobilized in the presence of sorbitol and subsequently incubated in growth medium retain the ability to bud (Fig. 2b). We did not quantify long-term immobilization and cell division, but we observe that cells stay immobile for hours and can divide while attached to the coverslip. We also tested the effect of an osmotic upshift by addition of 1 M sorbitol, which impacts the size and shape of *S. cerevisiae* (Fig. 3). Upon osmotic upshift yeast cells can lose up to 50% of their volume^24^. We observe that cells shrink within 90–130 sec upon addition of 1 M sorbitol. The recovery of the volume takes around thirty minutes. The cells kept completely immobile during and after their recovery (Fig. 3). We thus show that immobilization of yeast cells by APTES-glutaraldehyde allows for high-resolution imaging of cells.

**Figure 4:**
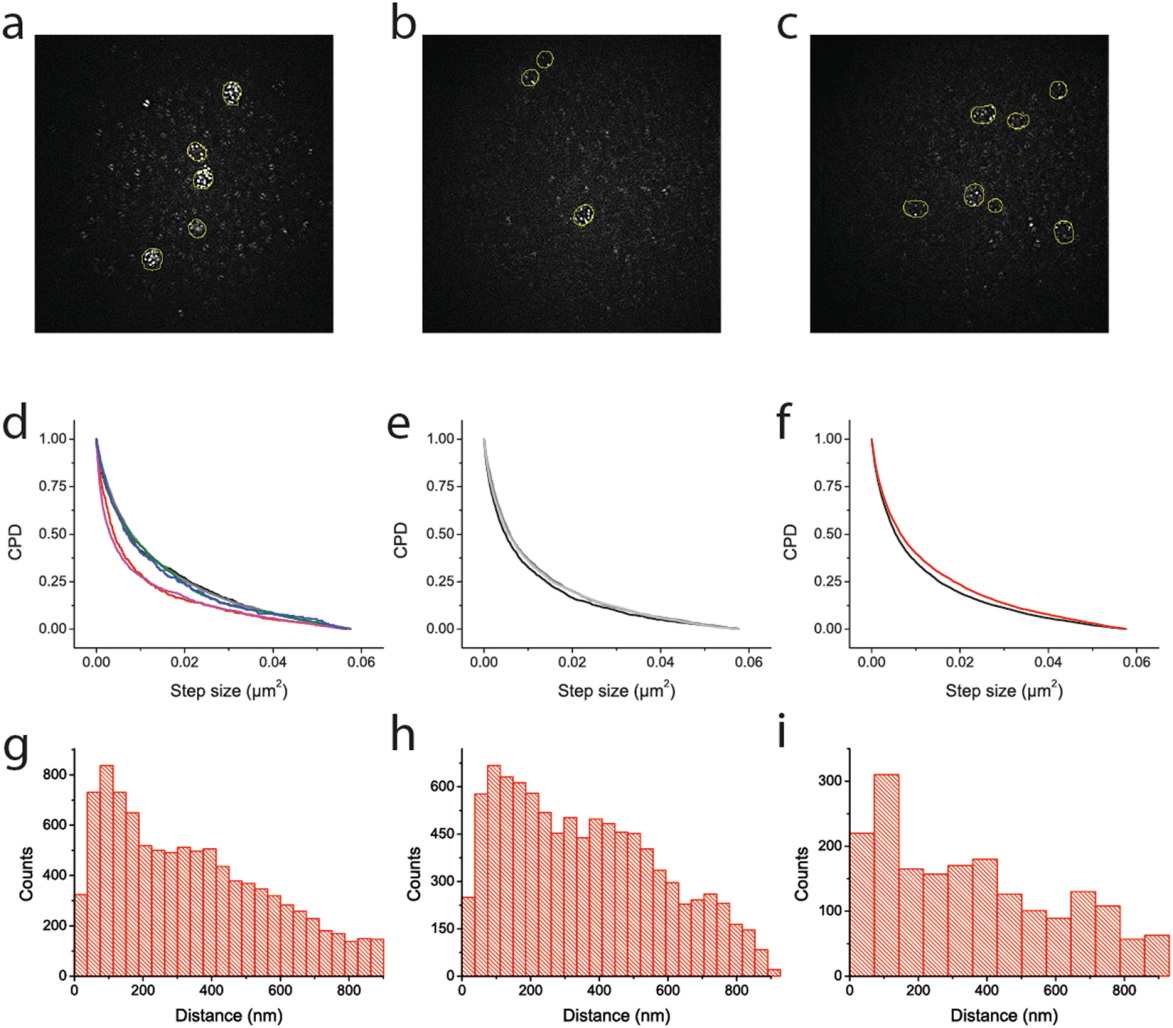
Comparison of *S. cerevisiae* cells immobilized by the ConA and the APTES-glutaraldehyde methods. Panels a–c show fluorescence background on the slides immobilized with ConA (**a**), glutaraldehyde (**b**) and EM-grade glutaraldehyde (**c**). A discoidal averaging filter (inner radius of 1, outer radius of 3 pixels; same as during the data analysis) was used on the images. Panels d–f show the cumulative probability distribution of step sizes of Can1-mCardinal molecules immobilized with ConA (**d)**; 6 independent experiments) or APTES-glutaraldehyde (**e**); 3 independent experiments). Panel f shows the cumulative probability distribution of all step sizes of Can1 in cells immobilized with ConA (red) or glutaraldehyde (black). Panels g–i show the distance distribution of localized molecules of Can1 to the center of the nearest eisosomes in cells. Panel g and h show the distribution of all Can1 found in cells immobilized with ConA (**g**) or APTES-glutaraldehyde (**h**). Panel i shows the sum of the molecules from the two independent experiments that have a much higher immobile population (red and pink lines on panel d).

We benchmarked the APTES-glutaraldehyde method against a ConA-coating, a commonly used method for immobilization of yeast^25,26^. We find that ConA-coating introduces increased background fluorescence when excited with a 561 nm laser, a wavelength which is popular for dual-color microscopy measurements in living organisms^27–29^. We imaged cells expressing Can1-mCardinal in TIRF mode (Fig. 4a–c) and find many more fluorescent foci outside of the cells with ConA than with APTES-glutaraldehyde immobilization; with the latter method the background depended on the quality of glutaraldehyde used, and in our hands, EM-grade glutaraldehyde from Sigma-Aldrich is preferred.

### Dual-color imaging and single-particle tracking

We performed a dual-color super-resolution microscopy experiment to determine if the fluorescence detected on ConA-immobilized slides has an impact on the tracking of individual molecules and compared the APTES-glutaraldehyde and ConA methods. We fused mCardinal to Can1 expressed from a plasmid under the control of the *gal* promoter. The mCardinal protein is relatively photo-stable, allowing us to track molecules for longer periods of times. The experiments were performed in TIRF mode to minimize the fluorescence background from interior of the cells. We tracked Can1-mCardinal over time and calculated the Cumulative Probability Distribution (CPD) of step sizes to determine the mobility of the protein (Fig. 4d–f). The CPD analysis shows two populations of molecules. The first population, which we call mobile, has a diffusion coefficient of 5.1 ± 0.8 and 5.4 ± 0.8 * 10^−4^ µm/s^2^ (mean ± SD) in cells immobilized with APTES-glutaraldehyde and ConA, respectively. The second population has a diffusion coefficient that is on the edge of our detection time, hence we refer to it as the immobile fraction. The mobile population consists of 57 ± 2% of the molecules in APTES-glutaraldehyde immobilized cells, which is in agreement with previous work^9^. In ConA-immobilized cells, the percentage is 59 ± 13%. Among the data sets collected with ConA-immobilized cells, we find two data sets showing a much higher percentage of immobile cells than the others, hence the larger standard deviation (Fig. 4d).

It has been shown by FRAP and SPT that Can1 is less mobile in and around the microcompartment of Can1 (MCC)/eisosomes^9,30,31^. To compare both immobilization methods in their applicability for localization microscopy, we now investigated the distribution of Can1-mCardinal with both protocols. We used Sur7-YPet as marker of MCC/eisosomes^32^ to determine the distance-dependence of the fraction of mobile and immobile Can1-mCardinal. Despite the difference in the fractions of immobile cells, the overall data sets show the same trends in Can1-mCardinal distribution relative to the MCC/eisosomes (Fig. 4g–i). We conclude that both immobilization methods report the same localization and lateral diffusion coefficient of mobile Can1-mCardinal, but the variation in mobile and immobile fractions is higher with ConA, most likely because part of the background fluorescence signal is taken as immobile protein.

### Immobilization of *E. coli*

**Figure 5:**
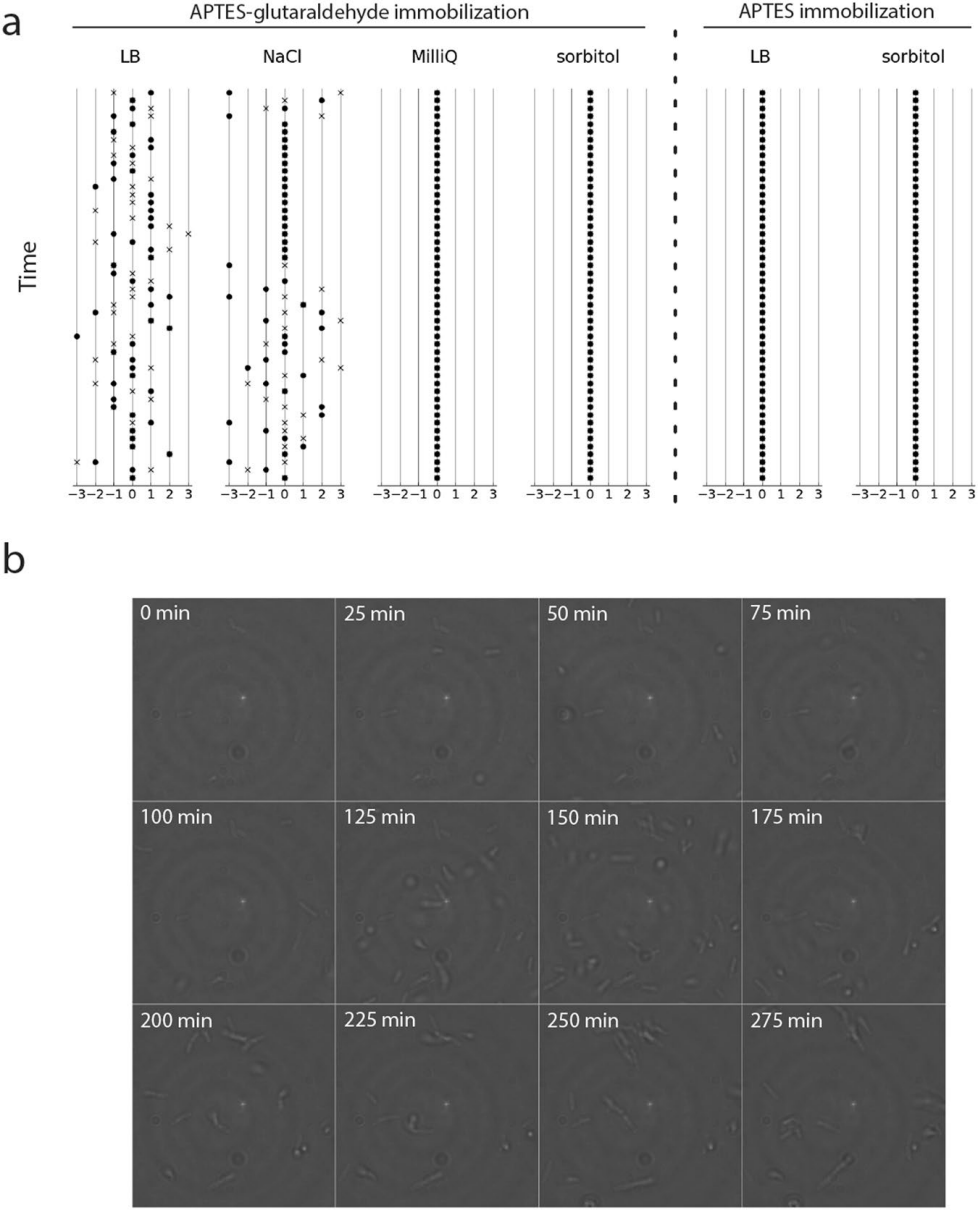
Immobilization of *E. coli* on APTES-glutaraldehyde- or APTES-treated slides. Panel a shows movement of the cells that were attached to the APTES-glutaraldehyde slides in the indicated solutions; the data are benchmarked against immobilization on APTES. The final concentrations in the attachment solutions are Lysogeny Broth (LB), 75 mM NaCl, milliQ or 150 mM sorbitol, as indicated at the top of the figure. The x-axis of the graphs shows the detected movement between two consecutive frames measured in pixels (Dots represent movement along x-axis, crosses along y-axis). Multiple frames are plotted on the y-axis. Panel b shows growth of *E. coli* in LB after immobilization of the cells in 150 mM sorbitol.

Next, we immobilized *E. coli* on APTES-glutaraldehyde coverslips. When low fluorescence background is needed, *E. coli* is usually immobilized on APTES-treated slides^33^. We performed immobilization experiments on APTES coverslips with and without glutaraldehyde treatment. As expected immobilization did not work in Lysogeny Broth (LB) as the attaching solution. We tested 150 mM sorbitol, 75 mM NaCl, and MilliQ as attaching solutions (Fig. 5a, Supplementary Movie 2). *E. coli* cells are completely immobilized when MilliQ or sorbitol are used, but we observe a lot of movement when cells are immobilized in NaCl. Thus, similar to what we saw with yeast, *E. coli* cells are effectively immobilized on APTES-glutaraldehyde coverslips when the attaching solution does not contain ions. Importantly, the cells attached to the slide still divide with a doubling time similar to that of free-floating cells (Fig. 5b).

### Immobilization of giant-unilamellar vesicles

We challenged our method further by immobilizing synthetic vesicles with dimensions between 20 to 50 µm. We formed phase-separating giant-unilamellar vesicles (GUVs) from a mixture of DPPC/DOPC/Cholesterol (4:3:3) lipids. As the method needs primary amines to react with glutaraldehyde, we added a small fraction (0.1 mol%) of DOPE to the mixture from which the GUVs are formed. Because GUVs are fragile it is not possible to remove the medium from the well and replace it with a different one or use solutions of very different osmolality. As the GUVs are formed in 200 mM sucrose solution, we decided to dilute the GUVs in 100 mM NaCl, 200 mM glucose, 200 mM sorbitol or 200 mM sucrose and thus prevent osmotic shocks (Supplementary Movie 3). We did not quantify the movement of GUVs because our method uses a transmitted-light image on which GUVs are not visible. GUVs were tracked by fluorescence only. The changes in background and diffusion of big lipid domains influenced the overall image, which precluded accurate measurements of GUV movement. Additionally, unbound vesicles could not be removed without collapsing most of the immobilized GUVs, at least not in the current set up of our measurements. Despite, the difficulties in quantifying GUV movement, we clearly observed a large fraction of immobile surface-bound GUVs on APTES-glutaraldehyde treated coverslips. Remarkably, we get the best results when the GUVs are diluted in 100 mM NaCl solution, which shows that the APTES-glutaraldehyde method works in the presence of relatively high concentrations of salt.

Next, we investigated the effect of the concentration of DOPE in the lipid mixture on the attachment of the GUVs to the coverslips. We prepared GUVs from mixtures containing 0.1 to 5 mol% of DOPE. Additionally, we tested a mixture of 2 mol% DOPE plus 2 mol% of methoxy(polyethylene glycol) derivatized 1,2-dioleoyl-sn-glycero-3-phosphatidyl ethanol amine (mPEG-2000-DOPE); mPEG-2000-DOPE has previously been reported to facilitate reproducible formation of large GUVs^34^. We do not see significant differences in the immobilization of the vesicles between the mixtures with different amounts of DOPE. The surface-bound GUVs are all immobile, and there is always some lipid debris on the slide.

**Figure 6:**
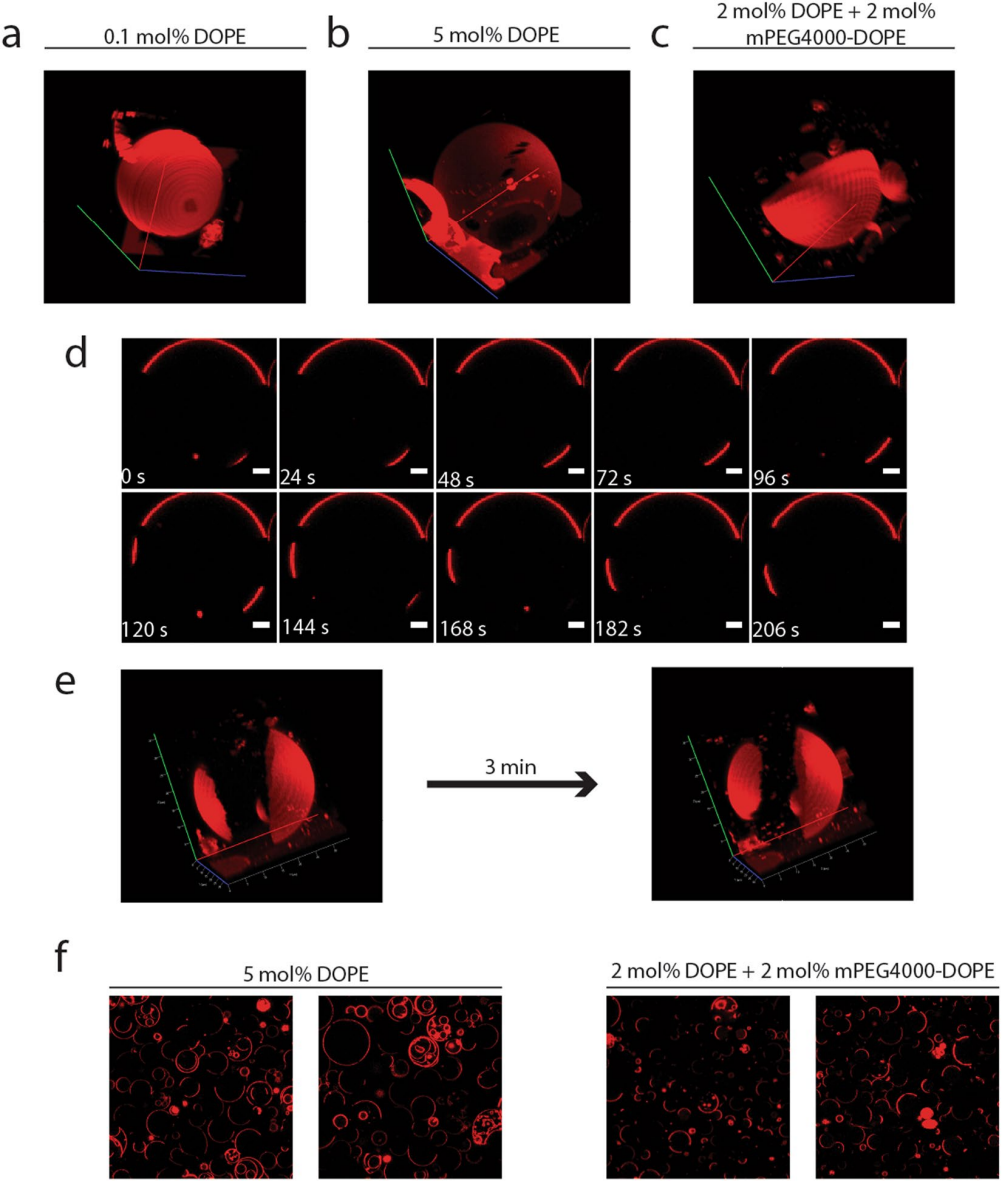
Immobilization of GUVs. Panels a–c show 3D images of GUVs composed of DPPC/DOPC/Cholesterol|(4/3/3) with addition of various amounts of DOPE: 0.1 mol% (**a**) or 5 mol% (**b**), or 2 mol% of DOPE plus 2 mol% mPEG4000-DOPE (**c**). Panels d and e show movement of large lipid domains in 2D over a period of 4 min (**d**); scale bar is 5 µm) and in 3D (**e**). Panel f shows the influence of mPEG4000-DOPE on the stability of the GUVs.

To determine if the vesicles are deformed when attached to the glass, we made 3D images of the GUVs (Fig. 6a–c). We found that the GUVs are almost completely spherical in all cases. We do not notice a preference for the liquid-ordered (L_o_) or liquid-disordered (L_d_) phase to be attached to the slide. While imaging GUVs attached to the glass we observed that regions in L_d_ phase diffuse through the L_o_ phase, but we did not quantify the speed of diffusion because the domains differed highly in size. We also observed fusion of the domains (Fig. 6d,e). Diffusion and composition changes of the lipid domains have previously been studied in free floating vesicles^35–37^. Our method of immobilization of GUVs eliminates the need to correct for drift by vesicle movement.

To further test the effect of mPEG2000-DOPE on the GUVs, we determined their stability using vesicles with 5% DOPE as benchmark. We find that after 24 h of storage at room temperature the mPEG-2000-DOPE GUVs still appear to be unilamellar, while the 5% DOPE vesicles became multilayered (Fig. 6f).

**Figure 7:**
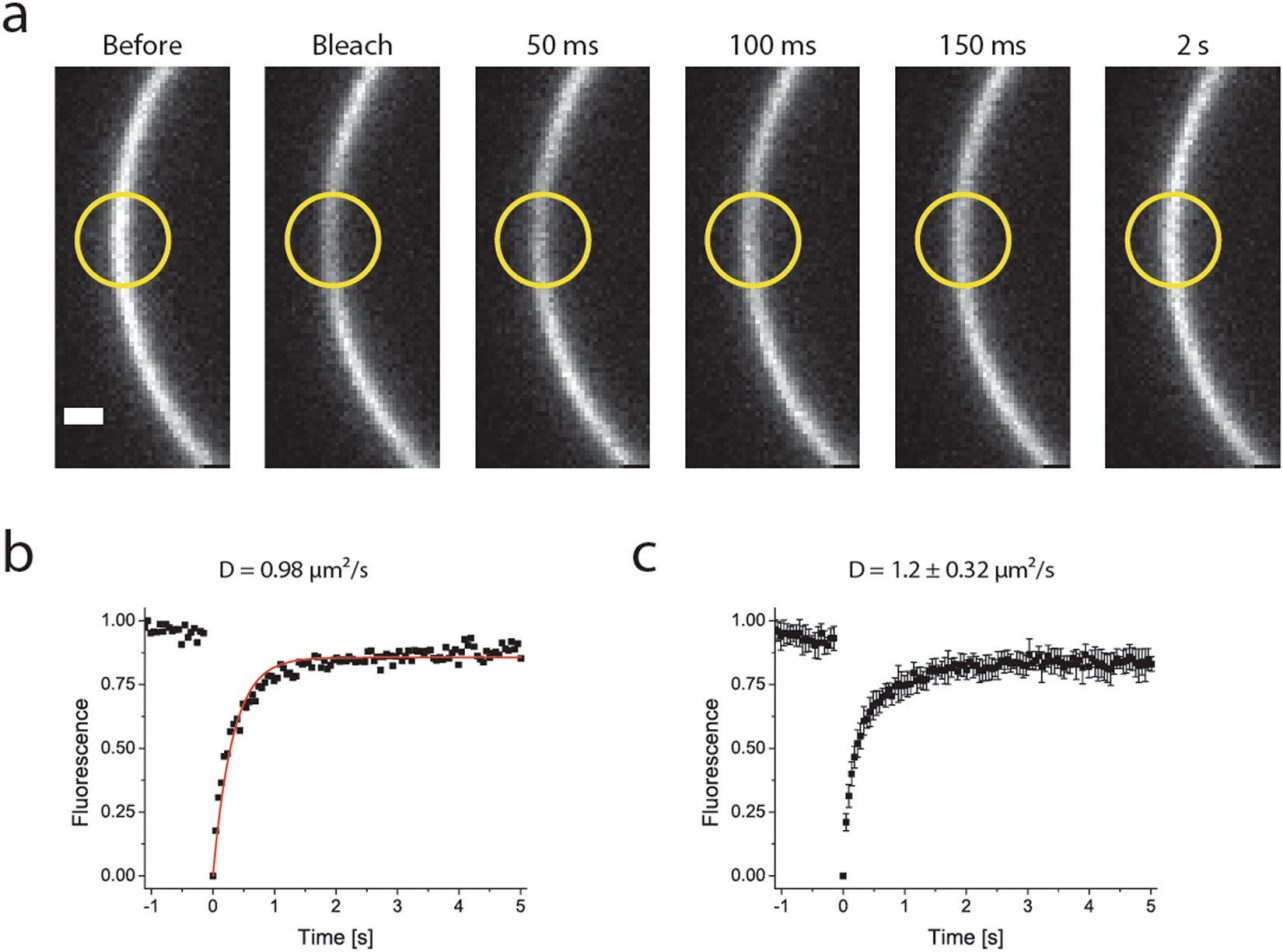
Lipid diffusion in the L_d_ domain of GUVs, probed by FRAP. Panel a shows part of the microscopy images. The yellow circle indicates the bleached area. Panel b shows normalized fluorescence within the bleached area over time (black squares) and the fit of the data (red line). Panel c shows the average and standard deviation of normalized data from several experiments (n = 8). Scale bar is 1 µm.

We performed fluorescence recovery after photobleaching (FRAP) experiments to determine the lateral diffusion of ATTO655-DOPE in the L_d_ phase of vesicles composed of DPPC/DOPC/Cholesterol (4:3:3) with 0.1 mol% of DOPE. The diffusion is extremely fast and close to the limit of what we can measure with our microscopy setup. We thus imaged only part of the GUVs to be able to accurately determine diffusion coefficients (Fig. 7). We found that 82 ± 2% of the fluorescence was recovered with a halftime of 196 ± 43 ms (mean ± SD). The diffusion coefficient, calculated from the halftime of recovery, is 1.2 ± 0.32 µm^2^/s, which is similar to the values previously reported for lipid diffusion in the L_d_ phase of free-floating phase-separating GUVs^38^. Thus, the APTES-glutaraldehyde method is suitable to determine the mobility of lipids (and presumably proteins) in the different domains of GUVs.

## Discussion

For cell immobilization in high-resolution fluorescence microscopy the method should (i) be easy and fast to implement; (ii) give rise to minimal background fluorescence; (iii) not affect the viability of the cells; (iv) allow analysis of the cells under (as close as possible to) native conditions; and, most importantly, (v) not allow for movement of the imaged objects. For yeast, ConA immobilization works well, but the background fluorescence can be problematic in TIRF measurements. The here described APTES-glutaraldehyde method proves versatile for different types of cells including synthetic lipid vesicles and gives reduced background fluorescence when compared with ConA.

For the testing of our method we used two commonly studied microorganisms, the eukaryote *Saccharomyces cerevisiae* and the bacterium *Escherichia coli*, as well as synthetic membrane systems. The yeast cell is surrounded by a cell wall that is composed of glucan, chitin and proteins that are extensively glycosylated^39,40^. The negative charge of the yeast cell surface is independent of pH^41^. *E. coli* is a Gram-negative bacterium that has an outer membrane (OM) and a lipopolysaccharide (LPS) layer exposed to the outer medium^42^. As most cellular cell surfaces, the OM proteins and the LPS are (mostly) anionic. Similar to yeast, we observed that the cells are not immobilized in the presence of salt, but the method works well with non-ionic attaching solutions. For reasons that are not clear, the APTES-glutaraldehyde method does not work for the immobilization of *S. cerevisiae* and *E. coli* in the presence of salt, possibly because primary amines needed for labeling are shielded under these conditions.

The presence of salt did not affect the immobilization of GUVs containing as little as 0.1 mol% DOPE. GUVs are important for biochemical and biophysical studies of lipid domain formation, molecule diffusion, protein function, protein-lipid interactions and others, and we here show that the APTES-glutaraldehyde is very suitable to study these processes when high sensitivity and minimal mobility of the vesicles are required. The GUVs were very stable on the coverslips and we did not detect major deformation of the vesicles.

In conclusion: we have developed a generic method for the immobilization of living and synthetic cells on surfaces that allow the structure of the cells and dynamic processes in the cell and cellular membranes to be studied, using both ensemble (FRAP) and single-molecule fluorescence microscopy techniques (super-resolution imaging and single-particle tracking). We benchmark the APTES-glutaraldehyde method against other known methods of cell immobilization. Importantly, we show that, even though the cells are immobile for hours, they are still capable of growing and dividing with rates comparable to that of non-immobilized cells.

## Materials and Methods

### Reagents and materials

We used glutaraldehyde from Sigma-Aldrich, product number 340855, and Electron Microscopy grade glutaraldehyde from Sigma-Aldrich, product number 49628; the latter showed lower background fluorescence. APTES (3-Aminopropyl)triethoxysilane) was obtained from Sigma-Aldrich, product number 440140. All other chemicals were reagent grade and obtained from various commercial sources. High precision coverslips (type #1.5 H) were obtained from Ibidi GMBH, product number 10812.

### Strains and growth conditions

A *S. cerevisiae* BY4742 derivative with Sur7-YPet integrated in the chromosome (Matα his3Δ1 leu2Δ0 lys2Δ0 ura3Δ0 sur7::SUR7-YPET), and carrying a plasmid with Can1-mCardinal under the control of the galactose promoter (pRS426GAL1-GFP; Can1 fused to mCardinal with 16 amino acid linker) was used^9^. The yeast cells were grown in synthetic drop-out media containing 2% [w/v] D-raffinose, without uracil, lysine and arginine but with 1 g/l of the dipeptide lysine-lysine present. The cells were grown for at least two days with dilution every morning and evening to keep them in the logarithmic phase of growth. Can1-mCardinal production in yeast cells was induced with 0.1% [w/v] D-galactose for 40 min before imaging. A milliliter of cell culture with OD_600_ of 0.6 was centrifuged for 4 minutes at 3000 *g* at room temperature. For attachment to the glass coverslips, the supernatant was removed and cells were suspended in a 150 mM sorbitol solution (unless specified otherwise, see Results).

*E. coli* MC1061 carrying a control plasmid with ampicillin resistance marker was used to immobilize the bacteria on various surfaces. The cells were grown in LB containing 100 µg/ml ampicillin. A pre-culture was grown overnight and subsequently the cells were kept in the exponential phase of growth by keeping the OD_600_ of the culture at ~0.5 by dilution with fresh media. Cells were centrifuged in a tabletop centrifuge (Eppendorf 5415D Centrifuge) in 1.5 ml tubes at 11,000 rpm for 2 minutes. For attachment to the glass coverslips, the supernatant was removed and cells were suspended in a 150 mM sorbitol solution (unless specified otherwise, see Results)

### GUV formation

GUVs composed of DPPC (1,2-dipalmitoyl-*sn*-glycero-3-phosphocholine), DOPC (1,2-dioleoyl-*sn*-glycero-3-phosphocholine) and cholesterol in 4:3:3 molar ratio with addition of 1:1000 of Atto655-DOPE (1,2-dioleoyl-*sn*-glycero-3-phosphoethanolamine) and ganglioside GM1, and varying (0.1 to 5 mol%) amounts of DOPE were formed by electroformation as previously described^38^. In short, lipid mixtures in chloroform:methanol (9:1) were placed on indium tin oxide (ITO)-coated plates (15 μl solution at a total lipid concentration of 5 mM) and dried under vacuum for 30 min. Next, the GUVs were formed upon hydration in 200 mM sucrose on the Vesicle Prep Pro (Nanion technologies), using a voltage of 1.1 V at 10 Hz for 1 h at 50 °C. After electroformation, the GUVs were cooled down to room temperature.

### Preparation and characterization of coverslips

First, high precision coverslips, 75 × 25 mm (0.17 mm thickness), were sonicated for 30 min at 30 °C in acetone (99.5%). They were washed 5 times with double-distilled H_2_O (ddH_2_O) and sonicated for 45 min at 30 °C in 5 M KOH. After sonication, the coverslips were washed several times with ddH_2_O. To remove all traces of KOH from the glass, the coverslips were sonicated for 10 min at 30 °C in ddH_2_O. The cover slips were washed an additional 10 times with ddH_2_O and once more with acetone. Residual solvents were removed by drying, using pressurized N_2_ and 30 min incubation at 110 °C. Next, the coverslips were plasma cleaned (PE-50, Plasma Etch, Inc) for 10 min. Directly after plasma cleaning, 2% APTES (v/v) in acetone was added to the coverslips. After 10 sec, the coverslips were washed three times with acetone and dried by using pressurized N_2_. The APTES coated coverslips were stored under vacuum and used within 3 days.

Before microscopy experiments, a bottomless µ-Slide (Ibidi, Inc) was placed on an APTES coated coverslip. To functionalize the amine of APTES with an aldehyde group of glutaraldehyde, 200 µl of 5% glutaraldehyde was added to each well. The amines were allowed to react with glutaraldehyde at room temperature for 30 min. After the incubation the glutaraldehyde was removed and the wells were washed 5 times with 200 µl of ddH_2_O. Then, 200 µl of cells in 150 mM sorbitol solution (unless specified otherwise, see Results) was added to the glass and incubated for 20 min. Sorbitol was chosen as a nonionic solution that is benign to the cells. After incubation the unbound cells were removed and the wells were washed 5 times with 200 µl of media specific for *S. cerevisiae, E. coli* or GUVs. Wells were left with 400 µl of media in them. We characterized the surface properties of the modified glass slides by contact angle measurements, using the OCA 15EC DataPhysics Instruments (GmbH). A drop of water with a total volume of 2 µl was put on top of the glass cover slips and images were taken.

### Imaging of *Saccharomyces cerevisiae*

A Home-built Olympus IX-81-ZDC inverted TIRF microscope was used. The microscope was equipped with a UAPON 1.49 NA ×100 TIRF objective (Olympus, Inc), a manual open-frame microscope stage (M-545) (Physik instruments, Inc), a 512 × 512 Electron Multiplying Charge-Coupled Device (EMCCD) C9100-13 camera (pixel size 80 nm, EM gain 1200×) (Hamamatsu, Inc) and Xcellence software (Olympus, Inc). Fluorescent proteins were excited with continuous wave (CW) lasers (Coherent Sapphire, Inc). During the microscopy experiments, Z-drift was compensated by z-axis control, which is an option built into the IX-81. The emission light was filtered using bandpass filters obtained from AHF (AHF, Inc). For imaging YPet^43^, the bandpass filter HC535/22 (Semrock, Inc) was used, whereas for imaging mCardinal^44^ the bandpass filter HC630/92 (Semrock, Inc) was used.

For both yeast and *E. coli* immobilization tests, time-lapse movies were obtained by illuminating the sample with white light and taking images every 100 ms for 50 frames. Quantification of the movement was achieved by selecting single cells and analyzing their position by correlating, using fast Fourier transforms, each pair of consecutive frames using in-house written software. The position of the pixel with highest intensity is determined and used to calculate x and y-movement of the cell.

In yeast, the localization and lateral diffusion of Can1-mCardinal were determined as described previously^9^. In short, time lapse movies were obtained by exciting the cells with a 561 nm laser every 10 seconds. The diffusion of molecules was estimated from their position in consecutive frames, and the cumulative probability distribution of step sizes was calculated^45^. The data yielded two populations of molecules, one assigned as “mobile” and the other as “immobile”. The lateral diffusion of Can1-mCardinal was determined relative to the MCC/eisosome marker Sur7-YPet, thereby obtaining a high-resolution (sub)structure of the cells in addition to information on the mobility of Can1. Super-resolution imaging was done by forcing YPet into photo-darkened state with a 514 nm laser and imaging subsequent stochastic blinking of the fluorophore. The distance to the centroid of the closest eisosomes was calculated for each of the Can1-mCardinal molecules.

The response of yeast to an osmotic upshift was tested by immobilizing cells on an APTES-glutaraldehyde modified coverslip with a bottomless µ-slide, and filling of the wells with 200 µl of the appropriate media. To observe the temporal changes in the cells upon the addition of 200 µl of 2 M sorbitol (final concentration of sorbitol of 1 M), time-lapse movies were recorded. The sample was illuminated with white light, and images were taken every 2 sec for 100 frames. For monitoring the recovery over longer periods of time, images were taken every minute for 40 frames.

### Imaging of giant-unilamellar vesicles

A commercial confocal microscope LSM 710 (Carl Zeiss MicroImaging, Jena, Germany) was used. The microscope was equipped with a C-Apochromat ×40/1.2 NA objective. Blue argon ion (488 nm) and helium-neon (633 nm) lasers were used. Time-lapse movies were obtained by imaging the field of view every second to check for movement of the GUVs. The 3D images of GUVS were obtained by creating a “z-stack” of images at different focal depths. The lowest image was always taken right below the glass, and the highest images were selected above the GUVs, which depends on the size of the vesicles (from a few µm to 50 µm) imaged. The difference in z-distance between frames was 0.45 µm. The laser power was chosen such that the signal was somewhere in the middle of the dynamic range of the APD detector. FRAP experiments were performed by imaging only a small area of the membrane of the GUVs to achieve an acquisition time below 50 ms. A spot with a diameter of 1 μm was bleached at high laser intensity, after which the attenuated laser was used to record images every 50 ms for 6 seconds; the pre-bleaching fluorescence was obtained from 20 images prior to the bleach. The halftime of recovery and lateral diffusion coefficients were calculated as in previous work^9^, which is based on work of Axelrod and colleagues^46^.

## Electronic supplementary material

Supplementary Video 1

Supplementary Video 2

Supplementary Video 3

## Data Availability

The datasets generated are available from the authors on request.
